# Bioengineered therapeutic systems for improving antitumor immunity

**DOI:** 10.1093/nsr/nwae404

**Published:** 2024-11-12

**Authors:** Ying Cao, Wenlu Yan, Wenzhe Yi, Qi Yin, Yaping Li

**Affiliations:** State Key Laboratory of Drug Research & Center of Pharmaceutics, Shanghai Institute of Materia Medica, Chinese Academy of Sciences, Shanghai 201203, China; School of Life Sciences, Jilin University, Changchun 130012, China; State Key Laboratory of Drug Research & Center of Pharmaceutics, Shanghai Institute of Materia Medica, Chinese Academy of Sciences, Shanghai 201203, China; University of Chinese Academy of Sciences, Beijing 100049, China; State Key Laboratory of Drug Research & Center of Pharmaceutics, Shanghai Institute of Materia Medica, Chinese Academy of Sciences, Shanghai 201203, China; University of Chinese Academy of Sciences, Beijing 100049, China; State Key Laboratory of Drug Research & Center of Pharmaceutics, Shanghai Institute of Materia Medica, Chinese Academy of Sciences, Shanghai 201203, China; University of Chinese Academy of Sciences, Beijing 100049, China; Yantai Key Laboratory of Nanomedicine & Advanced Preparations, Yantai Institute of Materia Medica, Yantai 264000, China; State Key Laboratory of Drug Research & Center of Pharmaceutics, Shanghai Institute of Materia Medica, Chinese Academy of Sciences, Shanghai 201203, China; University of Chinese Academy of Sciences, Beijing 100049, China; Yantai Key Laboratory of Nanomedicine & Advanced Preparations, Yantai Institute of Materia Medica, Yantai 264000, China; Shandong Laboratory of Yantai Drug Discovery, Bohai Rim Advanced Research Institute for Drug Discovery, Yantai 264000, China

**Keywords:** immunotherapy, biological organisms, gene engineering, synthetic biology, surface engineering

## Abstract

Immunotherapy, a monumental advancement in antitumor therapy, still yields limited clinical benefits owing to its unguaranteed efficacy and safety. Therapeutic systems derived from cellular, bacterial and viral sources possess inherent properties that are conducive to antitumor immunotherapy. However, crude biomimetic systems have restricted functionality and may produce undesired toxicity. With advances in biotechnology, various toolkits are available to add or subtract certain properties of living organisms to create flexible therapeutic platforms. This review elaborates on the creation of bioengineered systems, via gene editing, synthetic biology and surface engineering, to enhance immunotherapy. The modifying strategies of the systems are discussed, including equipment for navigation and recognition systems to improve therapeutic precision, the introduction of controllable components to control the duration and intensity of treatment, the addition of immunomodulatory components to amplify immune activation, and the removal of toxicity factors to ensure biosafety. Finally, we summarize the advantages of bioengineered immunotherapeutic systems and possible directions for their clinical translation.

## Teaser

This review presents an overview of bioengineered therapeutic systems based on cells/bacteria/viruses for cancer immunotherapy, with a particular emphasis on their engineering strategies and acquired functions.

## INTRODUCTION

Cancer immunotherapy aims to promote systemic immune responses to defend against tumors [1]. Current immunotherapies are based on the following strategies: (i) passive immunotherapy to directly combat tumors using effective components of the immune system, such as adoptive cell transfer therapy and cytokines; (ii) active immunotherapy to enhance essential immune response steps, such as vaccines and adjuvants; (iii) normalization immunotherapy to correct immune deficiencies, such as immune checkpoint-blockades (ICBs) [2]. Despite advances achieved in clinics, only a fraction of patients with advanced cancer profited from immunotherapy [3]. Besides the highly heterogeneous and mutable nature of tumor cells, the low drug delivery efficiency constrains the effect and safety of the therapy.

Cells and their derivatives have been used as vectors or active ingredients to enhance the efficacy and safety of immunotherapy against cancer due to their endogenous activities, low immunogenicity, homologous targeting capacity and prolonged circulation time. However, primitive cells cannot meet complex drug delivery demands because of shortcomings including limited biological activity, poor targeting efficiency, graft-versus-host disease (GVHD) and potential toxicity. For example, first-generation chimeric antigen receptor (CAR)-T cells rely solely on CD3ζ-mediated T-cell activation and lack intracellular co-stimulatory signals, resulting in an inability to achieve sustained expansion and maintenance of antitumor effects [4]. Bacteria exhibit significant antitumor activity *in vitro*; nevertheless, their low *in vivo* efficacy and dose-dependent toxicity limit clinical application. Oncolytic viruses remodel the tumor immune microenvironment and inhibit tumor growth by directly killing tumor cells, promoting cytokine secretion, inducing the release of tumor-associated antigens, and carrying genes that encode active proteins. Natural viruses with toxicity lack precision in tumor killing. Consequently, developing bio-derived therapeutic systems with optimized functions is essential.

**Figure 1.**
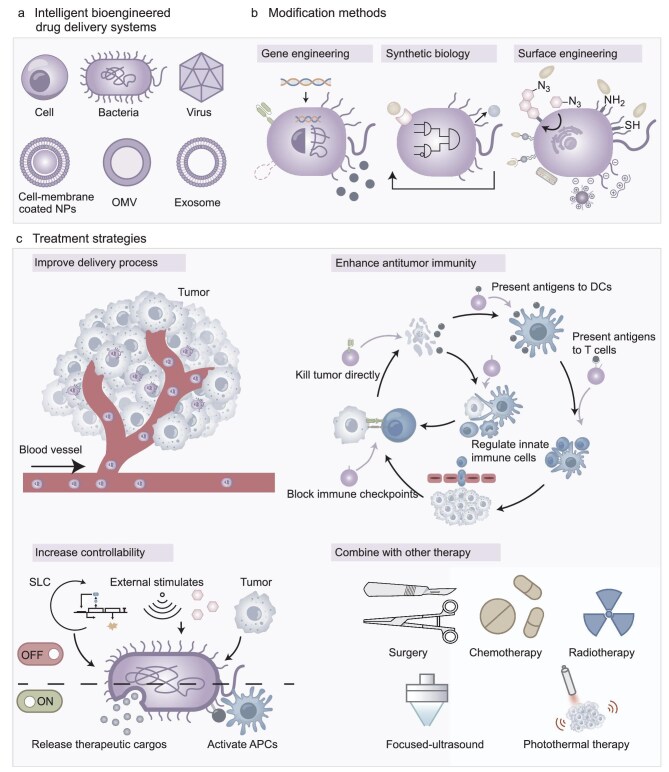
Scheme of bioengineered therapeutic systems for improving antitumor immunity. (a) Types of bioengineered therapeutic systems, which can be categorized as cells, bacteria, viruses and their derivatives. (b) Illustration of modification methods including gene engineering, synthetic biology modification and surface engineering. (c) Therapeutic strategies including improving delivery efficiency, enhancing anti-tumor immunity, increasing controllability and combining with other therapies. NPs: nanoparticles; OMV: outer membrane vesicles; DCs: dendritic cells; APCs: antigen-presenting cells; SLC: synchronized lysis circuit.

Recent advances in gene editing, synthetic biology and surface modification have made precise engineering of cells and bacteria possible as they can complement new functions for native cells and the development of novel cell-based therapies. The engineering strategies usually focus on two purposes, which can be defined as addition and subtraction: to endow cells with an intended ability (e.g. expressing cytotoxic agents) and/or to diminish undesired activity (e.g. deleting pathogenic genes). Herein, strategies employing engineered cells/bacteria/viruses for improving antitumor immunotherapy are summarized (Fig. 1). This review outlines the technologies for engineering cells/bacteria/viruses, comprising genetic engineering, synthetic biology and surface engineering. Their applications in modifying biological components, including cells, cell membranes, exosomes, bacteria, bacterial outer membrane vesicles (OMVs) and viruses, are elaborated to provide references for designing new cell/microorganism-based immunotherapy. At last, the constraints and prospects of this field are discussed.

## GENE-ENGINEERED ORGANISM-DERIVED SYSTEMS FOR ANTITUMOR IMMUNOTHERAPY

Inserting gene sequences into cells to introduce a new protein or overexpress an existing protein has the potential to endow the cell-derived systems with additional activity and targeting capacity for efficient drug delivery and enhanced antitumor immunotherapy [5,6]. Besides, gene editing can knock out specific gene sequences, thereby alleviating immunosuppression [7] or reducing the potential virulence of bacteria or oncolytic viruses [8]. Interestingly, manipulation of cellular or bacterial genomes may change the protein composition of their secretion products, obtaining engineered exosomes and bacterial OMVs. This section outlines commonly used genetic-engineering modification methods and summarizes strategies for designing genetically engineered biomimetic drug delivery systems (DDSs) for antitumor immunotherapy.

### Modification methods of gene-engineered organism-derived systems

Genetic engineering introduces genes constructed as hybrid DNA molecules into the recipient cells, resulting in alterations to the original genetic characteristics of the organisms and the creation of new required varieties.

#### Gene transfection methods

**Figure 2.**
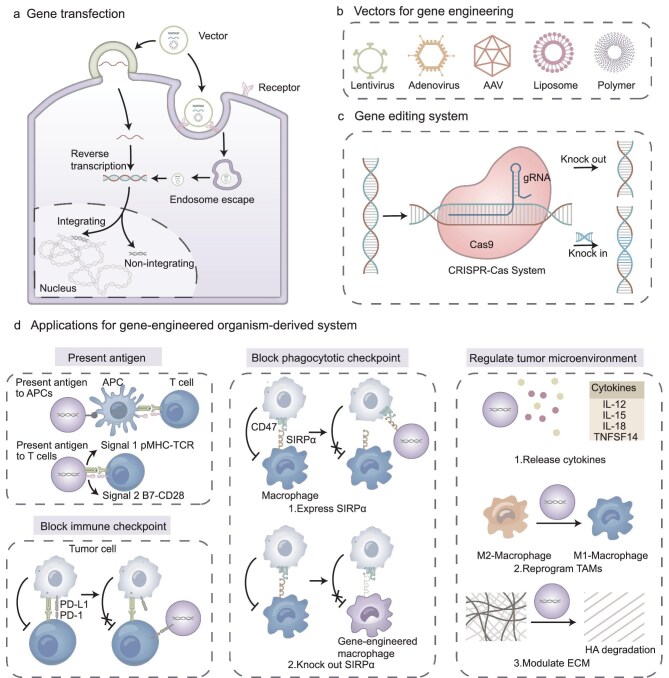
Gene-engineered organism-derived system. (a) Process of gene transfection. First, the gene vector enters the cell by membrane fusion or endocytosis. Inside the cell, the vector releases DNA or RNA fragments, and RNA is converted to DNA by reverse transcription. Depending on whether DNA is integrated into the genome of the transfected cell, transfection can be categorized as transient or stable. (b) Types of vectors used for gene transfection. (c) The CRISPR/Cas gene editing system consisting of gDNA and Cas that can knock out specific segments of the genome or introduce target genes into the genome. (d) Application of gene-engineered systems in tumor immunotherapy. Modified organism-derived systems enhance tumor immunotherapy through various mechanisms: delivering antigens to APCs or directly to T cells as tumor vaccines; expressing immune checkpoint molecules such as PD-1 on the surface to relieve tumor immunosuppression; blocking phagocytosis checkpoints by knocking out the SIPRα-related genes in macrophages or expressing SIPRα; regulating the tumor microenvironment by secreting cytokines, reprograming TAMs and degrading the ECM. AAV: adeno-associated viruses; CRISPR: clustered regularly interspaced short palindromic repeats; Cas: CRISPR-associated nuclease; APC: antigen-presenting cell; PD-1: programmed cell death protein 1; PD-L1: programmed cell death ligand 1; SIRPα: signal regulatory protein alpha; pMHC: peptide-major histocompatibility complex; TCR: T cell receptor; TNFSF: tumor necrosis factor super family; ECM: extracellular matrix; HA: hyaluronic acid.

Gene transfer into cells or bacteria can be achieved by virus or non-viral vector carrying the recombinant gene of interest (Fig. 2a), or by directly transferring plasmid through physical methods such as electroporation and microinjection [9].

Viral vectors include non-integrating vectors based on adenoviruses and adeno-associated viruses (AAVs) and integrating vectors based on lentiviruses and retroviruses. Viral vectors are widely used in tumor therapy because of their high efficiency. For example, adenoviral vectors are used in delivering tumor suicide genes and preparing oncolytic viruses clinically [10,11]. Lentiviral and retroviral vectors are commonly used in the construction of genetically engineered therapeutic cells. Lentiviral vector-based reprogramming CAR-T cells have been applied in clinical trials [12]. Additionally, lentiviral vectors are utilized for developing cell membrane- or extracellular vesicle-based immunotherapy systems [13,14]. Considering the safety concerns and fabrication costs of viral vectors, various synthetic non-viral vectors such as cationic lipids and polymers have been designed for gene transfection (Fig. 2b). Cationic liposomes or polymers, including poly-L-Lysine (PLL) [15], poly-ethylenimine (PEI) [16] and poly-amidoamine (PAMAM), can bind to DNA by electrostatic force to form a complex and improve gene uptake by the target cells [17].

#### Gene editing systems

Gene introduction can be divided into random integration of genes into the host cell genome and site-specific gene knock-in by gene editing systems. Clustered regularly interspaced short palindromic repeats (CRISPR)-associated nuclease (CRISPR/Cas), consisting of single guide RNA (sgRNA) and Cas proteins, is the most mainstream of the existing gene editing technologies (Fig. 2c). When the CRISPR/Cas system is delivered near the target gene, the sgRNA recognizes the target site and the Cas9 protein recognizes the protospacer adjacent motif (PAM) sequence adjacent to the target site, resulting in a double-strand break (DSB) specifically [18]. Cells can repair DSBs by two mechanisms, non-homologous end-joining (NHEJ) and homology directed repair (HDR), allowing gene knock-out and knock-in, respectively.

CRISPR/Cas systems can be used to downregulate the level of immune checkpoints to prevent immune escape of tumor cells [7], and reduce bacterial virulence by knocking out genes of pathogenic bacterial proteins [8]. Besides, they can also be used to insert immunoreactive molecules into the organisms for immune stimulation, such as introducing CD40 ligands to CAR-T cells or oncolytic viruses [19,20].

The efficiency of gene transfection is evaluated by analyzing protein expression profiles. Qualitative or semi-quantitative protein detection can be conducted with western blotting, immunocytochemistry and immunocytofluorescence assays. Quantitative detection of protein expression levels can be accomplished through the enzyme-linked immunosorbent assay (optimal for secreted proteins) and flow cytometry. It should be noted that for engineered cell derivatives such as OMVs, protein expression detection is required in both the source cells and their derivatives to avoid anomalies in the manufacturing process [21]. If the therapeutic agents or their vectors need to be encapsulated into the engineered cell membrane, the encapsulation can be qualitatively confirmed through transmission electron microscopy (TEM) observation, dynamic light scattering analysis and evaluation of zeta potential. In addition, the rate of drugs coated by cell membranes can be quantitatively measured by fluorescence-based detection [22].

### Application of gene-engineered organism-derived systems

Gene-engineered biologically derived systems with improved tumor targeting and antitumor potency can trigger innate and adaptive antitumor immune responses through multiple strategies.

#### Enhancing antigen presentation

Tumors can escape immune surveillance by downregulating antigenicity and antigen presentation efficacy [23]. Tumor-associated antigens, tumor cell lysates and tumor cell membranes can be utilized as tumor vaccines to facilitate antigen cross-presentation by antigen presenting cells (APCs). APCs carrying tumor antigens can also activate antitumor immune responses directly. However, immune activation via natural components is limited by inadequate antigen expression and the lack of co-stimulatory molecules.

The combination of genetic engineering and cell biological properties enables the controllable design of tumor antigen delivery systems (Fig. 2d). Genetic engineering can modify cells and their derivatives to co-deliver antigens and adjuvants to APCs [21,24]. Besides this, delivery systems based on cells expressing major histocompatibility complex (MHC) molecules and co-stimulatory molecules can act as artificial APCs (aAPCs) to stimulate T cells directly [25,26].

##### Delivering antigens to APCs.

Genetic engineering can add adjuvants or synthetic antigens to antigen delivery systems, enhancing the recognition and presentation of tumor-associated antigens by APCs and reducing potential toxicities of natural antigens [24,27].

Tumor-derived exosomes, possessing ample tumor antigens and satisfactory size to penetrate tumors, are widely used for antigen presentation. Gene editing enables exosomes to express proteins with specific affinity to bind therapeutic agents conjugated with protein ligands, potentializing them as an effective and flexible platform for delivering cargo. For example, a tumor antigen-adjuvant co-delivery system was constructed by conjugating biotin-labeled immune adjuvant cytidine-phosphate-guanosine (CpG) oligonucleotide onto the genetically engineered exosome expressing streptavidin fusion protein, enhancing tumor-specific responses compared to unmodified exosomes [27].

Genetic engineering can alter the type of antigens displayed by cell-derived delivery systems, such as adding neoantigens and tumor microenvironment (TME)-associated antigens. The nature of the antigen can also be adjusted to preserve antigenicity and delete pathogenicity. Fibroblast activating protein α (FAP) is an ideal mesenchymal target for solid tumor immunotherapy, yet the protein itself is not suitable for vaccine preparation because of its tumor-promoting effects. To address this problem, tumor-cell-derived exosome-like nanovesicles expressing a mutated FAP without hydrolytic activity were constructed, which prompted immune responses against tumor cells and cancer-associated fibroblasts [24].

Bacteria can also serve as vectors for tumor antigens. Chen *et al.* genetically engineered *Staphylococcus epidermidis*, a bacterium that lives on healthy skin, to express a tumor antigen. The live bacteria were colonized to the skin of tumor-bearing mice, stimulating the production of tumor-antigen-specific T cells without causing inflammation and infection [28]. Nanosized bacterial OMVs secreted by Gram-negative bacteria have the potential to activate APCs with their immunologically active components, including bacterial antigens and pathogen-associated molecular patterns (PAMPs) [29]. Employing genetic engineering techniques can disrupt the key genes for virulence factors such as lipopolysaccharide (LPS) synthesis and thus synthesize low-toxicity OMVs [30]. Gene engineering can also endow OMVs with tumor antigens on the surface or load them in vesicle lumen. Cheng and colleagues designed a ‘plug-and-play’ OMV tumor vaccine platform by binding tumor antigens onto the surface of OMVs via the interaction between the protein tags and catchers, which effectively delivered tumor antigens and achieved strong immune stimulation to combat tumors [21].

##### Delivering antigens to T cells.

APCs activate T cells through dual signal stimulation: (i) the first signal is provided by the binding of the MHC molecule-antigen complex with the T-cell receptor (TCR); (ii) the second signal is provided by the interaction between the co-stimulatory ligand of APCs with the co-stimulatory receptor of T cells, of which B7 (CD80, CD86)/CD28 and 4-1BB/4-1BBL are relatively important. Genetic engineering is conducive for constructing effective artificial antigen presentation systems by increasing the expression of MHC molecules and co-stimulatory factors on cells or cell membrane nanoparticles [31,32].

Tumor cells, presenting a certain degree of MHC I themselves, have the potential to activate T cells if artificially endowed with co-stimulatory molecules. For instance, nanovesicles derived from genetically modified tumor cells with CD80 overexpression provided the necessary dual signals to boost antitumor immunity mediated by CD8^+^ T cells. Harnessing the natural antigen-presenting ability of dendritic cells (DCs), the antigen self-presentation and immunosuppression reversal (ASPIRE) nanovaccines were designed with the membranes of DCs, which are genetically engineered to express tumor-specific peptide MHC-I and anti-programmed cell death protein 1 (PD-1) antibodies, with upregulated B7 levels after maturation. ASPIRE nanovaccines activated T cells, leading to robust antitumor responses that can prevent tumor growth, metastasis and recurrence [25]. Red blood cells are attractive for preparing engineered antigen-presenting systems because of their high biocompatibility and convenient fabrication [33]. In a study, the engineered red blood cells presenting antigenic peptides bound to MHC-I, co-stimulating ligand 4-1BBL and interleukin (IL)-12 were fabricated to directly drive the expansion of antigen-specific T cells with reduced immunotoxicity and genotoxicity [34].

#### Blocking the immune checkpoint

Multiple immune checkpoint inhibitors have been developed to reactivate T cells [35]. However, the clinical benefit of ICBs is moderate due to ineffectiveness and safety concerns. Targeted delivery of immune checkpoint inhibitors to tumor tissue provides a viable direction for improving ICB-based immunotherapy. Several engineered biologically derived platforms have been prepared to controllably deliver and release immune checkpoint inhibitors through a combination of genetic engineering and biomimetic technology (Fig. 2d). Programmed cell death ligand 1 (PD-L1) blockade nanovesicles could also encapsulate immunotherapeutic agents to trigger antitumor immunity jointly [36].

Crosstalk in the tumor immune microenvironment may dilute the effects of immune activation. For instance, while interferon (IFN) promotes CD8^+^ T cell expansion, it also inevitably upregulates multiple immune checkpoint molecules. An engineered T-cell membrane-coated nanoparticle presenting PD-1 and encapsulating an IFN epigenetic inducer, as well as an engineered OMV expressing the PD-1 extracellular domain, were created to counteract the negative feedback regulation of IFN [6]. In contrast to nanovesicles, live cell-based delivery systems can self-proliferate and differentiate at the targeted site. Engineered long-lived plasma cells and hematopoietic stem cells prepared with the CRISPR/Cas9 system and lentivirus transfection respectively could secrete anti-PD-1 antibodies sustainably, and have potential for long-term immunotherapy [37,38]. For modifying non-nucleated, terminally differentiated cells, genetic engineering can be operated on their precursor cells. For instance, to leverage the capacity of platelets that can accumulate in surgery wounds, Zhang *et al.* procured PD-1-expressing platelets from genetically modified megakaryocytes, thereby reinvigorating depleted CD8^+^ T cells [39]. The strategy of blocking PD-1/PD-L1 with genetically engineered cell membranes can be extended to other immune checkpoints. The binding of CD155 expressed on tumor cells to T-cell immune receptors with immunoglobulin and immunoreceptor tyrosine-based inhibitory motif domains (TIGIT) present on immune cells, results in the suppression of immune cell function [40]. Construction of cell membrane vesicles expressing TIGIT for delivering chemotherapeutic agents synergistically increases antitumor immunotherapeutic effects [41].

#### Blocking the phagocytotic checkpoint

Revitalizing innate antitumor immunity provides another direction for durable and multilevel tumor control. The most widely studied phagocytic checkpoint is the CD47-signal regulatory protein α (SIRPα) pathway [42]. To block CD47 on tumor cells, an exosome was genetically engineered to express a high-affinity variant of SIRPα for CD47 surface binding at a minimal quantity, initiating phagocytic clearance of cancer cells (Fig. 2d) [43]. Similarly, an engineered SIRPα-variant-presenting cell membrane-coated magnetic nanoparticle was fabricated to reprogram tumor-associated macrophages (TAMs) and inhibit the CD47-SIRPα pathway, resulting in synergistic activation of the macrophage immune response and controlled tumor progression [13].

#### Regulating the tumor microenvironment

During tumor development, the TME promotes tumor growth, angiogenesis and metastasis by secreting immunosuppressive cytokines and recruiting immunosuppressive cells. Several genetically engineered delivery systems have been developed to target the TME, with specific strategies including reversal of macrophage polarization, release of cytokines and modulation of the extracellular matrix (ECM) (Fig. 2d).

Cell-derived delivery systems have been used to target the TAMs and increase the proportion of M1-type macrophages with antitumor activity. Cell-derived exosomes loaded with biotherapeutic agents via genetic engineering provide promising platforms to enhance the stability and delivery efficiency of immunologically active drugs [44]. For instance, myeloid cells with the ability to target pre-metastatic niches were transfected with lentivirus to express IL-12. The IL-12-secreting cells reversed the immunosuppression in the pre-metastatic niche and reduced tumor metastasis [5]. Bacteria and viruses, as natural immune activators, can also be engineered to secret antitumor cytokines. Hyaluronic acid (HA) existing in a dense ECM can be degraded by hyaluronidase to loosen the matrix and boost drug penetration. Thomas *et al.* prepared a genetically engineered *Escherichia coli* Nissle (EcN) strain to over-generate OMVs in the tumor matrix. These OMVs released recombinant hyaluronidase to reduce the HA content of the ECM, thereby increasing the penetration of antibodies and immune cells [45].

In conclusion, genetic engineering is a versatile means of organism modification, offering required functions beyond wild-type cells. Additionally, the expression levels of genetically modified markers must be evaluated to guarantee the stability of the genetic modifications. The targets can be selected based on one or more nodes in the process of activating innate and/or acquired immune responses. The combination of multiple targets may produce synergies. The prospective advancement of gene editing toolkits and more discoveries with regard to anti-tumor immune molecular mechanisms will facilitate the deployment of genetically modified cells.

## SYNTHETIC-BIOLOGY-ENGINEERED ORGANISM-DERIVED SYSTEMS FOR ANTITUMOR IMMUNOTHERAPY

Synthetic biology aims to reprogram cellular behavior and create cell-based synthetic systems by introducing genetic circuits into cells. Genetic circuits can be typically deconstructed into three major modules: the input module sensing and converting external signals to endogenous signals; the manipulation module processing the endogenous signals and directing the output signals; and the output module altering cellular behavior according to the output signals. Genetic circuits can be categorized into open- and closed- loops based on whether the output signal gives feedback to the input signal [46].

Compared to conventional genetic engineering of living organisms, synthetic biology emphasizes the controllability and sensitivity of therapeutic responses through the following strategies: (i) introducing switch elements into the gene circuits to make the output responsive to timing and intensity; (ii) introducing tumor-responsive elements to achieve tumor-specific output. This section summarizes key strategies in synthetic biology focused on improving cellular immunotherapy, drug-carrying engineered bacteria and oncolytic virus therapy.

### Improving cellular immunotherapy

**Figure 3.**
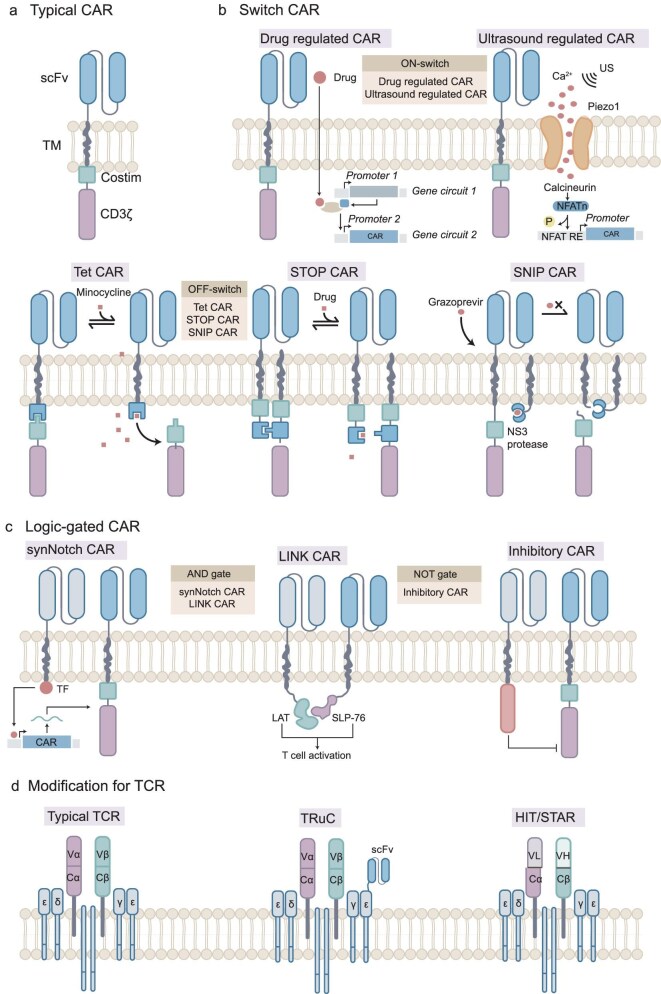
Synthetic biology engineering improving cellular immunity. (a) The typical CAR structure is comprised of three distinct domains: an extracellular domain, a transmembrane domain and an intracellular domain. (b) Introducing switch structures into CAR-T systems allows for the rational control of treatment intensity. The switch systems can be categorized into ON switch and OFF switch. In an ON-switch system, exogenous small molecules or physical stimuli activate corresponding gene circuits within the system, thus enabling the transcription and expression of CAR genes. In an OFF-switch system,exogenous stimuli alter the conformation of the CAR, inactivating it either temporarily or permanently. (c) Logic-gated CARs can accurately recognize tumor antigens and avoid off-target effects. The AND gate produces activated CARs only when two tumor antigens are present simultaneously. NOT-gated CAR-T cells have both effector and inhibitory CARs on the surface of the cells. When encountering normal cells expressing tumor-associated antigens, inhibitory CAR binds with normal cells and prevents the action of effector CAR. (d) Modification of TCR improves antigen recognition. The unmodified TCR can only recognize pMHC. Fusion of antibody structural domains with TCR subunits yields TruCs, enabling effective recognition of tumor surface antigens. Similarly, synthetic receptors such as HITs and STARs, which fuse immunoglobulin heavy and light chains (V_H_ and V_L_) to TCR-Cα and TCR-Cβ, can conduct antigen signaling in an MHC-independent manner. CAR: chimeric antigen receptor; TM: transmembrane domain; US: ultrasound; SNIP CAR: signal neutralization by an inhibitable protease CAR; synNotch: synthetic Notch receptor; LINK CAR: logic-gated intracellular network CAR; pMHC: peptide-major histocompatibility complex; TF: transcription factor; TruC: TCR fusion constructs; HIT: human leukocyte antigen-independent TCRs; STAR: synthetic TCR and antigen receptors.

CAR-T therapy has produced remarkable clinical responses and evolved to a third generation by introducing co-stimulatory signaling domains to promote cell proliferation, extend lifespan and enhance activity (Fig. 3a). However, it still has poor results in solid tumors and may cause cytokine release syndrome. Synthetic biology has updated CAR-T therapies by introducing user-defined sense-and-respond gene circuits that can respond to stimuli. Similarly, introducing synthetic gene circuits into mammalian cells could expand their sense range of intracellular or extracellular signals and produce more desired responses [47].

#### Improving controllability of therapeutic responses

A well-engineered therapeutic cell system, such as CAR-T therapy, necessitates an ON- or OFF- switching element to regulate therapeutic responses, thereby preventing the side effects of over-activation and ensuring effective treatment.

The activation of the ON-switch depends on the dosage of the inducer, thus inhibiting the cytokine release syndrome produced by over-activation (Fig. 3b). Chemical small molecules, mechanical forces and light can master the expression of CAR by introducing respective gene circuits, providing a safe and controllable strategy for precise immunotherapy. The ON-switch can also control the release of therapeutic cytokines loaded by the cells [48].

The OFF-switch is deactivated after sensing an external signal (Fig. 3b). The deactivation mechanisms are as follows: (i) incorporating inducible caspase suicide genes into CAR-T cells for permanent elimination when administering certain signals [49]; (ii) using small molecules to disrupt the antigen recognition of dimerization CAR and achieve temporary inactivation [50]; (iii) labeling CAR structures with induced degradation tags and recruiting small molecule controllers to initiate targeted proteasomal degradation [51]. Inducible silence of CAR can also be achieved by introducing small molecules, such as lenalidomide and grazoprevir, to induce repression of the gene circuit [51,52]. The inactivation is reversible as CAR activity is rejuvenated upon a reduction in the concentration of the small molecule controller.

#### Improving targeting effects

Poor targeting limits the application of CAR-T therapies against solid tumors. The extra-tumoral expression of the targets for CAR-T leads to off-tumor-on-target effects against healthy tissue. The irregular vascular system and dense ECM limit the infiltration and penetration of therapeutic cells into solid tumors. Therefore, synthetic biology-based design of CAR-T cells with tumor targeting and deep penetration can potentially improve the safety and efficacy of therapy.

To differentiate the influence of CAR-T therapy on tumors compared to normal tissues, gene circuits utilizing boolean logic gating including AND gate and NOT gate have been constructed (Fig. 3c). Activating AND-gated CARs requires at least two types of tumor antigens. For instance, Srivastava and colleagues engineered cells with an ‘if-then’ circuit by co-expressing synthetic Notch receptors (synNotch) and CAR receptors on the cell surface [53]. Specifically, the CAR combats tumor-associated antigen A only if the synNotch receptor recognizes tumor-associated antigen B. Inspired by synNotch, synthetic membrane proteolysis receptors (SNIPRs) with adjustable sensing and transcriptional regulation ability were designed from top down [54]. However, the off-target toxicity remains a concern of SynNotch or SNIPR, as they may kill normal cells expressing antigen A once activated by antigen B. To avoid the bystander-killing effect, the logic-gated intracellular network (LINK) CARs are designed, which implement a simultaneous dual-antigen AND gated response circuit for specific elimination of double-positive tumor cells [55].

NOT-gated CAR-T cells express both activating CARs paired with tumor-associated antigens and inhibitory CARs paired with ligands on the surface of non-tumor cells to ensure the safety of the treatment. The toxic effects of this CAR-T cell will be inhibited when it encounters non-tumor cells through the binding of the inhibitory CAR with the ligands on the surface of the innocent cells [56]. Similarly, Li *et al.* designed dual-targeted NOT-gated CAR-natural killer (NK) cells with active CARs that recognized tumor-associated antigens and inhibitory CARs that recognized NK cell markers, avoiding NK cell depletion caused by fratricide [57].

Gene circuits can also be introduced into other therapeutic cells to sense stimulus from changes in endogenous diseases and achieve tumor-specific effects. Fussenegger's team modified HEK-293T cells and human mesenchymal stem cells using a cell-contact sensing device. Once the engineered cells recognized target cells, a fusion protein structure on the membrane surface of engineered cells (CD43ex-45int) separated from the cell–cell interface due to external forces, activating the Janus kinase-signal transducer and activator of transcription (JAK-STAT) pathway and turning on the downstream therapeutic signaling expression [58]. Genetic circuits typically produce therapeutic proteins by activating transcriptional and translational pathways. The process of taking effects and responding to external signals may exhibit excessive lateness. To address this issue, Ye *et al.* developed a protease-based rapid protein secretion system (PASS). T cells engineered with the antigenic PASS identified tumor antigens and then released granzyme B and perforin to trigger apoptosis in target cells rapidly [59].

To test the efficiency of genetic circuit modification, quantitative real-time polymerase chain reaction detection was employed to quantify RNA expression, while the detection of the expression of target proteins utilizes similar methods to those in genetic engineering.

#### Improving antigen recognition

Plenty of work has been dedicated to preparing hybrid receptors that combine the advantages of both TCR and CAR (Fig. 3d). For instance, the T cell antigen coupler (TAC) is composed of an antigen-binding domain, CD3-binding domain and co-receptor domain. TAC redirects T cells to select antigens in a TCR-dependent and MHC-independent manner, ensuring a more controllable T-cell response [60]. Similarly, TCR fusion constructs (TruCs) fusing antibody structural domains to CD3 subunits also reprogram TCR complexes to efficiently recognize tumor surface antigens [61]. In addition, synthetic TCR and antigen receptors (STARs), as well as human leukocyte antigen (HLA)-independent TCRs (HITs), which both bind antigen-recognizing regions to TCR-constant regions, are capable of HLA-independent antigen recognition and TCR signaling [62,63].

Synthetic biology enables the use of cells other than T cells, such as induced pluripotent stem cells (iPSCs), as a source for preparing CAR-T cells, reducing the difficulty of CAR-T manufacturing. iPSC-derived CAR-T cells are expandable and exhibit a reduced propensity for immune rejection [64]. Furthermore, CAR-T cells derived from iPSC exhibited high therapeutic efficacy and prolonged action duration by genetically transducing with IL-15/IL-15Rα and knocking out inhibitory signals [65].

### Modifying bacteria to deliver cargos

Synthetic biology compensates for shortcomings of natural bacteria via multiple strategies including attenuating virulence, modifying motility, improving targeting and loading drugs that can be released in a controlled manner.

#### Enhancing responsiveness to tumors

**Figure 4.**
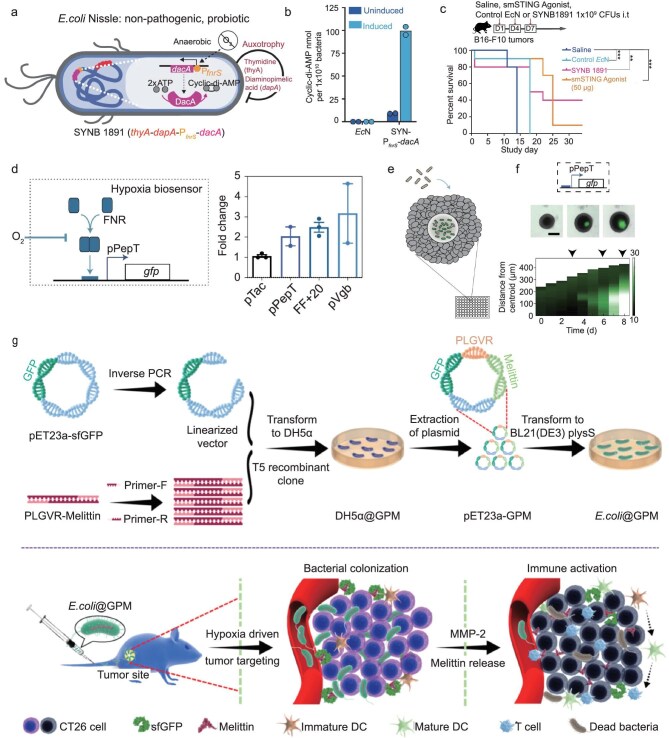
Increasing tumor responsiveness of engineered bacteria. (a) A hypoxia-responsive engineered bacteria, SYNB1891, designed for antitumor immunotherapy. (b) Under anaerobic conditions, cyclic di-adenosine monophosphate (cyclic di-AMP) abundance from bacterial cell pellets increases. (c) Tumor-bearing mice treated with SYNB1891 show attenuated tumor growth [66]. Copyright 2020, Springer Nature. (d) The hypoxia biosensor uses the pPepT promoter that relies on dimerized fumarate and nitrate reduction regulatory protein (FNR) to drive gene expression under low oxygen levels. (e) Engineered bacterial biosensors were co-cultured in tumor spheroids and monitored for biosensor activation. (f) Representative images of biosensors in tumor spheroids and corresponding space-time diagram demonstrating radially averaged fluorescence intensity of hypoxia (pPepT) biosensors [67]. Copyright 2021, Springer Nature. (g) Engineered bacteria enter tumors driven by hypoxia and release melittin in response to tumor matrix metalloproteinases [68]. Copyright 2023, American Chemical Society.

Through the introduction of genetic circuits, tumor hallmarks (such as poor oxygenation and low pH) can be combined with bacterial behavior (such as drug release and proliferation) to improve tumor targeting. An EcN strain with a hypoxia-inducible promoter and genes related to the synthesis of stimulator-of-interferon-gene (STING) agonists was designed to activate APC specifically in tumor sites (Fig. 4a, b) [66]. Meanwhile, chassis bacteria triggered the complementary immune pathway, resulting in significant antitumor activity (Fig. 4c). SYNB1891 performed well in a clinical trial (NCT04167137) for the treatment of advanced malignancies. Hypoxia-conditioned promoters as well as hypoxia- and acid-conditioned promoters linked by AND logic gates could also serve as switches in bacterial growth-related gene pathways to enhance bacterial localization in tumors (Fig. 4d, f) [67].

Genetic circuits that enable bacteria to express or secrete TME-responsive prodrugs could avoid the leakage of toxic agents. Xu *et al.* developed engineered EcN to deliver a cell-killing peptide, melittin (Fig. 4g) [68]. To avoid off-target toxicity, the melittin was present as an inactive promelittin, and once the engineered bacterium homed to the tumors, promelittin was cleaved by matrix metalloproteinases (MMPs) and transformed into the active melittin, killing surrounding tumor cells. Similarly, an MMP-responsive neoantigen was tethered on the surface of attenuated *Salmonella typhimurium* to recruit and activate immune cells in tumor sites [69].

#### Constructing a synchronized lysis circuit

Bacteria have a quorum-sensing system that detects the levels of particular signaling molecules in the surrounding environment [70]. Quorum-sensing gene circuits can automatically regulate the output of therapeutic proteins by sensing the bacterial colony density, circumventing drug-dose-related toxicity and protecting healthy organs.

**Figure 5.**
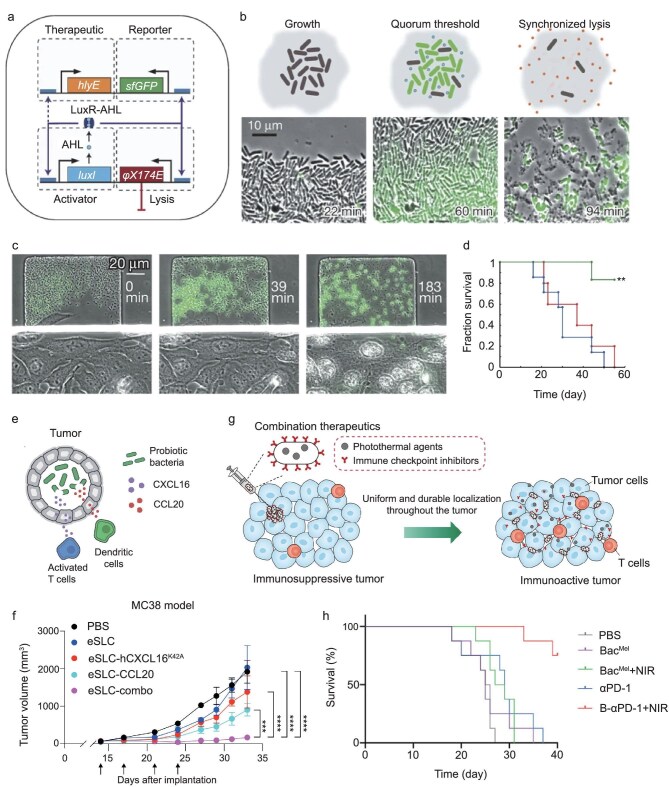
Improving controllability of engineered bacteria. (a) Schematic of an SLC: when the population density reaches the threshold at a critical autoinducer (AHL) concentration, the luxl promoter initiates the transcription of the downstream lysis module. (b) Bacteria with SLC undergo three processes in the lysis cycle: growth—reaching critical concentration—lysis. (c) Frames from the co-culture time series sequentially visualizing *S. Typhimurium* firing, lysis and HeLa cell death. (d) Fraction survival over time for the mice with hepatic colorectal metastases fed with the SLC-3 strains (blue), injected with 5-FU chemotherapy (red) or a combination of the two (green) [71]. Copyright 2016, Springer Nature. (e) Engineered bacteria containing SLC can release chemokines locally, and (f) slow tumor progression [74]. Copyright 2023, The American Association for the Advancement of Science. (g) Schematic of the engineered bacteria utilized for photothermal and ICB combination therapy. (h) Survival curves of orthotopic 4T1 tumor-bearing mice after different treatments [78]. Copyright 2021, Wiley‐VCH GmbH.

Din *et al.* designed a synchronized lysis circuit (SLC) to achieve drug release *in situ* by sensing the density of bacterial population colonized in the tumor core (Fig. 5a) [71]. The engineered bacteria colonized the tumor core and fabricated drugs until a quorum threshold was reached, at which point the bacteria lysed to release the drug (Fig. 5b). The periodically released drugs ultimately prolonged the survival of mice bearing hepatic colorectal metastases (Fig. 5c, d). To improve the safety of anti-CD47 therapy, an engineered *E. coli* strain containing SLC was developed for local and pulsed release of CD47 antagonists [72]. Similarly, engineered bacteria with SLC can be used to deliver immune checkpoint inhibitors to boost the tumor killing effects of T cells, and chemokines to recruit antitumor immune cells (Fig. 5e, f) [73,74]. Despite the advantages mentioned above, more research is needed to address the problem that mutant bacteria will evolve and evade proliferation-lysis circulation under selection pressure.

#### Improving controllability

Synthetic biology can also improve the controllability of therapeutic bacteria. External inducers, including chemical molecules and physical cues, can activate customized genetic circuits in engineered bacteria to perform complex and diverse functions.

Chemical molecules such as isopropyl-β-D-thiogalactopyranoside (IPTG) and arabinose often act as effectors to activate bacterial operons. In one example, Harimoto and co-workers developed an engineered bacteria with an IPTG-inducible capsular polysaccharide (iCAP) system to control the synthesis of CAP [75]. The administration of IPTG following the systemic administration of engineered bacteria resulted in the temporary expression of CAP on the bacterial surface, thereby protecting the bacteria from immune system clearance. Furthermore, the iCAP system of tumor-colonizing engineered bacteria could be activated *in situ* to enhance circulation and facilitate translocation to distal tumors.

Small molecules can also be used to initiate load secretion. Engineered bacterial systems with an arabinose-inducible promoter could secret OMVs containing tumor antigens *in situ* triggered by arabinose [76]. The controlled release of tumor antigens prevented immune tolerance caused by long-term antigen stimulation, thus effectively inhibiting tumor growth, metastasis and recurrence.

Physical stimulation with high specificity can modulate the engineered bacteria in a non-invasive manner. One strategy based on physical cues is to design temperature-sensitive engineered bacteria. Focused ultrasound-responsive engineered bacterial systems were designed to synthesize and release immune checkpoint inhibitors upon sensing localized heating [77]. Another strategy for physical modulation is to combine engineered bacteria with photothermal therapy (PTT) to construct light-sensitive engineered bacteria. Wang *et al.* designed a dual-engineered strain that stably expresses melanin as a photothermal agent while anchoring immune checkpoint inhibitors on the surface (Fig. 5g, h) [78]. Modification of EcN containing a thermosensitive tumor necrosis factor-α (TNF-α) gene circuit with gold nanoparticles to mediate photothermal conversion also enabled PTT-induced immunotherapy [79].

### Programming oncolytic viruses

To enable oncolytic viruses to infect tumor cells specifically and induce tumor-associated antigen release, two strategies are commonly utilized: (i) deletion of viral genes associated with virus replication whose function can be compensated by tumor cells; (ii) introduction of effector gene circuits that can be activated only by tumor-specific molecules.

#### Virus selective replication

Abnormally growing tumor cells upregulate some proteins associated with viral growth. Therefore, deletion of the genes of these proteins in the virus allows the virus to selectively replicate in tumor cells. Oncolytic vaccinia virus with the IL-21 gene recombined and the thymidine kinase gene deleted infected tumor cells more effectively than normal cells, acting synergistically with CAR-T immunotherapy [80]. Vaccinia viruses with the vaccinia growth factor or vaccinia protein B18R deleted also reduced the virulence to normal tissues because of their inability to replicate in normal cells without RAS overexpression or their susceptibility to clearance by immunocompetent cells [81].

Oncorine, the first oncolytic virus approved for clinical use, achieves selective lysis of tumor cells by deleting the coding region of the virulence protein that inactivates p53 in normal cells, resulting in the virus replicating only in malignant cells with abnormal p53 function [82]. The first Food and Drug Administration (FDA)-approved oncolytic virus T-Vec is a herpesvirus (HSV) that replaces the ICP34.5 gene (neurotoxicity factor) with the human colony-stimulating factors (GM-CSF) gene. ICP34.5 inhibits antiviral response pathways in normal cells, which are disrupted in tumor cells. Thus, T-Vec without ICP34.5 will be eliminated by normal cells and specifically infect tumor cells. Tumor cells can also be more susceptible to virus infection because of natural immune deficiencies (e.g. defects in the STING signaling pathway) [83].

#### Gene selective expression

Selective infection by oncolytic viruses can be achieved by inserting promoters or transcription factors that are more active in tumor cells. The cell-cycle-inhibiting adenoviral protein E1A can be specifically expressed under the control of prostate-specific antigen promoter [84], telomerase reverse transcriptase promoter [85], E2F1 promoter [86] and hypoxia-inducible transcription factor (HIF)-1α [87], which are upregulated in tumor cells. Conditionally replicating oncolytic adenoviruses CG-0070 and ONCOS-102 with GM-CSF genes and tumor-selective E2F promoter-driving E1A viral gene expression have been demonstrated to achieve clinical benefits.

Clinically used oncolytic HSVs delete the ICP34.5 gene, which may lead to a limited immune response. Liang *et al.* designed an HSV called CAN-3110 containing the ICP34.5 gene controlled by the glioblastoma-overexpressed nestin promoter, which demonstrated safety in a phase I clinical trial treating 41 patients with high-grade gliomas (NCT03152318). Tumor lysis induced by CAN-3110 recruited immune cells to the tumor and reversed the immunosuppressive TME in glioblastoma, providing a biological rationale for converting cold tumors to hot tumors [88]. Besides, microRNA (miRNA) responsive target elements were introduced to the gene circuits to control the replication and virulence of the viruses through degrading the transcripts required for viral replication when binding to miRNAs highly expressed in normal cells [89]. The introduction of AND logic gates into the genetic circuit facilitates more precise control of viral activity. SynOV1.1, a lysogenic adenovirus with a synthetic genetic circuit consisting of an α-fetoprotein promoter and miRNA responsive target elements that control specific replication in alpha-fetoprotein (AFP)-positive tumor cells, has been approved by the FDA [90].

Synthetic biology can be considered as the advanced version of genetic engineering. In the existing findings, it provides the ability to regulate the intensity, timing and environmental conditions of treatments with engineered cells, thereby facilitating the development of advanced therapeutic modalities based on genetic circuits. Nevertheless, this field is still in its infancy. Its ultimate goal is not to modify but to create cells according to therapeutic purposes. Therefore, genetic circuits should be precise enough so that the designed cells can behave in a way that corresponds to the desired therapeutic outcomes, even in the heterogeneous *in vivo* environments of diverse patients. In the future, computer-aided design tools may be employed to support the design of genetic circuits and to establish treatment regimens based on synthetic biology therapeutic strategies.

## SURFACE-ENGINEERED ORGANISM-DERIVED SYSTEMS FOR ANTITUMOR IMMUNOTHERAPY

Surface engineering aims to endow cell membranes with new functions by modifying them with advanced materials. Surface engineering strategies are currently classified into gene editing, chemical reactions, physical interactions and bioconjugation. In contrast to gene editing, discussed previously, the other techniques decorate cell membranes without altering the genetic information, which may be safer and easier.

By modifying molecules with targeting capacity or conjugating drugs with environment-sensitive linkers on cell membranes, cell-derived systems can deliver drugs precisely. Anchoring immune-normalizing drugs or immune-enhancing agents can confer added immunomodulatory activity to the delivery system. Furthermore, attaching other therapeutic reagents to the delivery system can achieve combination therapy.

### Modification methods of surface engineering

#### Chemical methods

Biomaterials can covalently bind to functional groups of biomolecules including proteins and glycans present on cellular and bacteria surfaces. However, reactions to existing moieties have low reaction rates and off-target risks [91]. In recent years, click chemistry methods have emerged to construct bioorthogonal reactions, which enable precise cell modification.

##### Covalent binding to existing groups.

**Figure 6.**
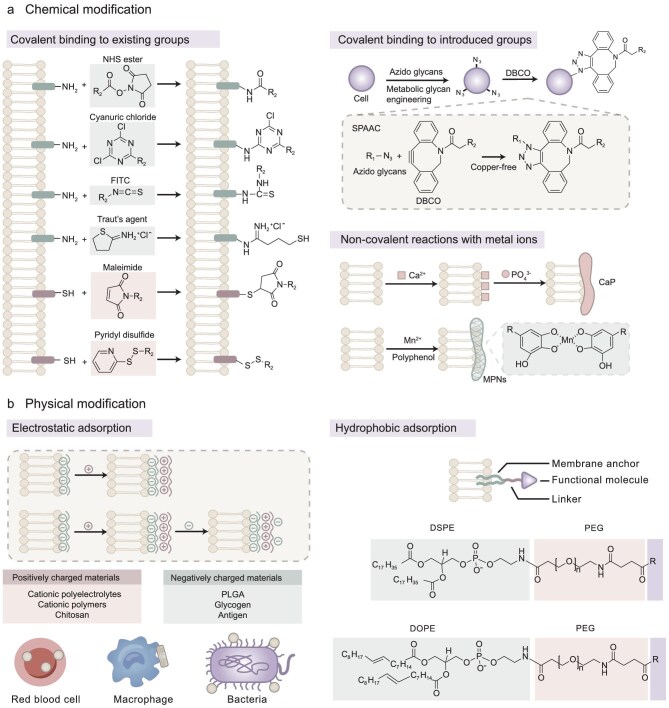
Schematic illustration of surface engineering. (a) Chemical modifications. Small molecules can form stable covalent bonds with amino and thiol groups present on the cell membrane surface. In addition, metabolic glycan engineering introduces azide groups to the cell surface, allowing for the reaction of DBCO and other specimens through copper-free click chemistry. The third type of chemical modification is the non-covalent binding of metal ions to the cell membrane surface, which results in the formation of a bionic mineralized layer or a metal polyphenol network. (b) Physical modification. Cationic materials can be bound to the surface of cells or bacteria by electrostatic adsorption. Typical examples of electrostatic adsorption include erythrocytes hitchhiking, macrophage backpacks and bacteria adhering to nanoparticles. Physical modification can be achieved by hydrophobic interaction-mediated membrane anchoring as well. Membrane anchoring complexes consist of a lipophilic anchor, a hydrophilic linker and the functional molecule. The commonly used membrane anchors are DSPE and DOPE, conjugating with a PEG linker. DBCO: dibenzocyclooctyne; SPAAC: strain-promoted alkyne-azide cycloaddition; MPNs: metal polyphenol networks; PLGA: poly(lactic-co-glycolic acid); DSPE: 1,2-distearoylsn-glycerol-3-phosphoethanolamine; DOPE: 1,2-dioleoyl-sn-glycero-3-phosphoethanolamine; PEG: polyethylene glycol.

Materials can be covalently linked to reactive terminal groups such as amine and thiol groups on the cell surface. The primary amine group (–NH_2_), which is located at the N-terminus of polypeptide chains and in lysine, can react with many chemical reagents including cyanuric chloride, N-hydroxysuccinimide (NHS) esters and fluorescein isothiocyanate (FITC) (Fig. 6a) [92]. For example, a biotinylated reagent with an NHS ester group can react with primary amines on the cell surface to form biotinylated cells [93]. Similarly, the NHS moiety can bind to the surface of Salmonella VNP20009 for the conjugation of heptamethine cyanine dyes [94]. Free thiols (–SH) can cross-link with most types of reactive groups. Chemicals that contain maleimide groups undergo Michael addition reaction with cell surface thiols, forming stable and irreversible thioether bonds [95]. In addition, pyridyl disulfide groups can form reducible disulfide bonds with thiols, enabling reversible covalent coupling of delivery components to the cell surface [96]. Notably, the amino groups can also be converted to thiols by thiolation reagents such as Traut's reagent for further decoration [97].

##### Covalent binding to introduced groups.

Click chemistry reactions can be used for highly selective chemical modifications without interfering with other biochemical reactions *in vivo*. Strain-promoted alkyne-azide cycloaddition (SPAAC) is the most commonly used click chemistry method, through which the azide group is added to the cyclooctyne, typically dibenzocyclooctyne (DBCO), without the need for copper (Fig. 6a) [98].

Metabolic glycan engineering is the most common strategy to endow cell membranes with azide groups. Specifically, cells are co-cultured with synthetic monosaccharides, such as N-azidoacetylmannosamine-tetraacylated (Ac_4_ManNAz), which contain azide groups. These sugar derivatives can be processed through metabolic pathways upon entering the cell, and finally presented as a glycoprotein on the cell surface [99,100]. With the aid of metabolic glycan engineering, azido groups are introduced onto the cell surface of T cells, DCs and NK cells, resulting in efficient bioorthogonal reactions for the coupling of biomaterials such as polyethylene glycol (PEG), antibodies, targeted peptides and liposomes [101–105].

The chemical modification efficiency of cells can be evaluated through diverse characterization techniques. For instance, TEM allows for the direct observation of the binding of nanoparticles to cells, while confocal microscopy facilitates the observation of the location of fluorescent dye-labeled molecules or groups and cells. Additionally, high-performance liquid chromatography can determine the loading ratio of chemical molecules [94–96]. Given that chemical binding may influence cell activity, it is essential to conduct further examination of cell proliferation and functions.

##### Non-covalent reactions with metal ions.

Biomimetic mineralization strategies create mineralized shells on the cell surface via coordination reaction. First, acidic residues on the cell surface chelate calcium ions, providing ‘nucleation sites’ for calcium phosphate (CaP). With time, calcium ions accumulate around the nucleation sites, which leads to the spontaneous crystallization of calcium phosphate. Eventually, calcium phosphate crystals form spherical aggregates that encapsulate the cells through ordered assembly [106] (Fig. 6a). Another strategy for modifying organisms with metallic materials involves the use of metal polyphenol networks (MPNs). Metal ions and phenolic ligands can quickly bind to the cell membrane through ligand interactions (Fig. 6a), forming robust nanocloaks to preserve the cellular protein component and enhance internalization [107].

#### Physical methods

Glycoproteins and lipids on the surface of cell membranes serve as ligands for physical interactions including electrostatic interactions, van der Waals forces, hydrogen bonding and hydrophobic interactions. Physical modification parts are mild, allowing one-pot preparation of the DDS.

##### Electrostatic adsorption.

Positively charged materials, including cationic polyelectrolytes, cationic polymers and chitosan, can be adsorbed directly onto cell membranes via electrostatic interactions [108–110]. Cell surface modification strategies based on electrostatic adsorption are derived into two models: the hitchhiking and the backpacking strategies (Fig. 6b). Erythrocytes with a double-sided concave structure and long circulation time are classical hitchhiking carriers. Nanoparticles are adsorbed on the cell surfaces through hydrophobic and electrostatic interactions followed by the diffusion of the membrane around the nanoparticles to strengthen adhesion [111]. Macrophages with the potential to target tumors are selected as nanoparticle backpack carriers. To avoid phagocytosis by macrophages, disk-shaped and high-aspect-ratio nanoparticles are harnessed for adhesion to the cell surface [112].

Negatively charged materials can be modified on the membrane surface through layer-by-layer adsorption [113]. For instance, glycol chitosan can be adsorbed onto the negatively charged surface of *E. coli* firstly, switching the surface charge to positive. Subsequently, the negatively charged poly(lactic-co-glycolic) acid (PLGA) nanoparticles can be adsorbed onto glycol chitosan [114]. Similarly, chitosan and PAMAM dendrimers function as intermediates to help bacteria adsorb negatively charged nanoparticles (Fig. 6b) [109,115].

##### Hydrophobic adsorption.

Phospholipids are amphiphilic molecules with long-chain aliphatic hydrocarbon chains at the tail, which can be inserted into the cell membrane by hydrophobic interactions. Phospholipid derivatives, such as 1,2-distearoylsn-glycerol-3-phosphoethanolamine-N-(polyethylene glycol)_2000_ (DSPE-PEG_2000_) and 1,2-dioleoyl-sn-glycero-3-phosphoethanolamine (DOPE)-PEG, with a high modification efficiency and little impact on the cellular phenotype, can be used to attach small molecule drugs, aptamers, peptides and polysaccharides on the cell surface (Fig. 6b) [8,116–119].

Generic characterization techniques based on spectroscopy, chromatography and electron microscopy can be employed for qualitative and quantitative analysis of the efficiency of physical modifications. Furthermore, the measurement of the surface potential of the cell membrane allows for determining the level of electrostatic adsorption.

### Application of surface-engineered organism-derived systems

#### Improving delivery efficiency

Organism-derived DDSs face multiple biological barriers. Drugs upon intravascular administration may undergo clearance by the mononuclear phagocyte system (MPS), enzyme degradation and the barriers formed with vascular endothelium, dense tumor ECM and targeted cell membrane [120]. Surface engineering can improve their *in vivo* behaviors via multiple strategies such as prolonging the circulation time of the platforms and enhancing their tumor targeting, accumulation and penetration capabilities.

##### Stealth in circulation.

A temporary masking layer on the surface with exogenous components may improve the stability and pharmacokinetic properties of biologically derived delivery systems. Ideal modifying masks must be stable in circulation and dissociate at the target location. Intravenous OMV administration triggers a systemic inflammatory response. To address this issue, Qing *et al.* covered calcium phosphate shells on the surface of OMVs. The outer shell shielded OMVs from immune system recognition during systemic circulation and dissolved in response to the acidic TME, thereby exposing the immunogenic OMVs in order to exert immune reprogramming efficacy [106]. In a separate study, oncolytic adenovirus was encapsulated in biomineralized and pyranose oxidase-engineered OMVs to promote adenovirus accumulation in the tumor region and enhance tumor autophagy [121]. Additionally, attaching PEG on CAR-T cells through copper-free click chemical modification reduced monocyte interaction and alleviated immune-related side effects [100].

##### Enhancing targeting effect.

To construct biomimetic delivery systems with targeting ability, ligand molecules with specific affinity to marker proteins on target cells are modified on delivery systems by surface engineering techniques.

Small molecules, typically less than 1 kDa, have simple chemical structures and are easy to synthesize. Folic acid is used in targeting strategies as it can bind strongly to its receptor, which is overexpressed on the cancer cell membrane. Kim *et al.* anchored folic acid to the surface of NK cells using lipids, thus enhancing the recognition of cancer cells by NK cells [122]. Polysaccharides are a common class of protein ligands as well. Neu5Acα2-6Gal*β*1-4GlcNAc trisaccharide modification of NK cells by metabolic glycan engineering could direct specific killing of lymphoma B cells with CD22 expression [123]. HA, as a ligand for the tumor marker CD44, can also be decorated on the surface of NK cells to enhance tumor cell targeting by lipid insertion or click chemistry [95,118].

Aptamers are single-stranded oligonucleotides with a specific 3D structure that enables them to recognize targets with high affinity [124]. Shi *et al.* designed CD30-binding aptamers and anchored them to the surface of NK cells for enhancing the targeting of CD30^+^ lymphoma cells by NK cells [125]. Yet, the degradation of aptamers in biological media also needs to be taken into account.

Protein-based biomolecules, such as monoclonal antibodies, are frequently utilized as targeting agents due to their stable binding to paired antigens. In a study, cetuximab was attached to the surface of NK cells through metabolic glycan engineering to target colon cancer and inhibit tumor growth [105]. Functional peptide fragments can also be used for surface modification. The Neuropilin-1 targeting peptide, RGERPPR (RGE), was used to modify the exosome surface via click chemistry. This modification allowed exosomes to cross the blood-brain barrier and target gliomas [99].

#### Relieving immune resistance.

Surface engineering of biologically derived DDSs by adding molecules that alleviate tumor immunosuppression can normalize antitumor immunity.

Drugs that reprogram M2-type TAMs to M1-type, such as metformin and Toll-like receptor (TLR) agonists, were attached to the surface of the cell-derived systems to promote pro-inflammatory factor secretion and affect other immune cells [126]. Furthermore, it is possible to modify TAMs directly by constructing adhesive backpacks on their surface for continuous release of pro-inflammatory factors, maintaining the M1-polarized TAM phenotype [108,110]. Coupling phagocytic checkpoint inhibitors to the surface of the delivery system is also a promising strategy for reversing immunosuppression. Nie *et al.* constructed exosomes from M1-type macrophages modified with CD47 and SIRPα antibodies via click chemistry reaction to abrogate ‘don't eat me’ signaling and reprogram TAMs [101].

Platelets represent a promising source of carriers for immunotherapeutic drugs given their capacity to target surgery wounds and their extensive physiological functions. Wang *et al.* obtained antibody-armed platelets by covalently coupling aPD-L1 to the platelet surface, which can actively aggregate to the surgical bed, alleviating tumor recurrence and metastasis after surgery [127]. Building on this, the same team developed another combined cellular drug delivery strategy based on platelets through click chemistry and covalent modifications for the treatment of acute myeloid leukemia [128]. In this delivery strategy, hematopoietic stem cells function as guides and are coupled to drug-conjugated platelets via click chemistry. When reaching the bone marrow under the guidance of hematopoietic stem cells, platelets release aPD-1 and enhance the anti-leukemia immune response. This cell combination drug delivery strategy is expected to be used in the treatment of diseases other than leukemia, inspiring therapeutic cellular therapies.

Elevated levels of lactate and adenosine, along with hypoxia, are abnormal tumor metabolism markers that intensify immunosuppression and are chosen as targets for enhancing immunotherapy. A recent study utilized a biomimetic mineralization strategy to modify manganese dioxide coatings on bacterial surfaces, which alleviated the hypoxic microenvironment of tumors and reversed tumor immunosuppression by virtue of manganese dioxide's ability to catalyze oxygen production from hydrogen peroxide [129].

#### Amplifying immune activations

Surface-engineered DDSs can amplify antitumor immunity by enhancing DC maturation and antigen presentation as well as promoting T cell activation by DCs. To improve antigen uptake and processing by DCs, Ji and colleagues attached antigen peptide via click chemistry and coupled the innate immunostimulant CpG via lipid anchoring to the surface of engineered influenza A virus (IAV) expressing anti-PD-L1 nanobodies for lung-targeting delivery of vaccines [130]. Wang *et al.* modified attenuated *Salmonella* with cationic particles on the surface, which can electrostatically adsorb tumor antigens and efficiently presented antigens to DCs through flagellar motility [109].

Surface engineering enables the creation of aAPCs. Erythrocytes serve as chassis cells for aAPCs due to their high surface area and flexibility. One study obtained aAPCs by chemically coupling MHC-containing antigenic peptides and CD28 co-stimulatory factors on the surface of erythrocytes to directly activate circulating T cells [93]. Varying the number and density of antigen particles coupled to the surface of erythrocytes enables targeted delivery of antigens [111]. To enhance DCs adhesion to T cells, Yang *et al.* utilized click chemistry to covalently attach glycopolymers to the surface of DCs, resulting in improved T cell activation [131]. Xiao *et al.* constructed nano aAPCs based on DC membranes with peptide-MHC complexes, B7 and anti-CD3ε antibodies on the surface through metabolic glycan engineering [104]. This aAPC provided multiple signals for T cell activation, inhibiting the growth of melanoma and colorectal cancer. To improve the T-cell-based therapies, therapeutic agents can be modified on T cells by surface engineering. Tang *et al.* constructed the bioreducible T cell agonist IL-15-loaded nanogel as a backpack of the CAR-T cell. The nanogel was decorated with PEG-PLL and anti-CD45 antibodies to enhance affinity with T-cell membranes and inhibit T-cell endocytosis, and released IL-15 in response to T-cell activation [132].

#### Synergizing with other therapies

Living cell systems, such as tumor cells with homologous targeting ability, immune cells that invade a location responding to chemokines, and anaerobic bacteria that tend to colonize hypoxic tumors, have the potential to deliver drug cargos to tumor sites. For instance, chemotherapeutic agents or photosensitizers can hitchhike to tumor sites after being anchored on cell membranes or bacteria and achieve synergistic antitumor immunotherapy combined with bacterial immunogenicity or cellular immunity.

Tumor cells overexpressing adhesion molecules have the potential to be developed as DDSs after excluding their oncogenicity, for example, by treatment with liquid nitrogen [133]. Triple-negative breast cancer cell cadavers, conjugated with anti-PD-1 antibodies and doxorubicin (DOX)-loaded liposomes using small-molecule chemical cross-linkers, could target tumor metastases in the lungs through adhesion proteins on their surfaces [134]. The ternary composite ingredients led to synergistic activation of antitumor immune responses, ultimately inhibiting lung metastasis. In addition, chemotherapeutic agents can be encapsulated in surface engineered cells as well. For instance, macrophages modified with LPS, an immune adjuvant, through receptor-ligand binding were further loaded with DOX for targeted drug transportation [135]. LPS reprogrammed TAMs towards the M1-type and induced the release of TNF-α, which enhanced the antitumor effect of DOX.

As programmable ‘robot factories’, bacteria, modified with different therapeutic reagents on the surface, can deliver drugs according to specific needs and exert synergistic effects of combination therapies. Bacteria can carry drugs through simple chemical coupling. *Salmonella* biohybrid coupled with the PD-L1 inhibitor JQ1 and the photosensitizer N782 mediated robust photo-immunotherapy through bacterial immunogenicity, immune checkpoint inhibition and photothermal therapy-induced release of tumor antigens [94].

Compared to genetic engineering, surface engineering offers a more accessible solution for the introduction of foreign molecules onto the cell surface, which can help to provide the cells with a variety of functions. However, the biocompatibility of the introduced molecules needs to be considered, as well as the selection of appropriate reaction types to avoid damaging cellular activity. Techniques for the precise determination of the degree of surface modification and the separation of cells with different degrees of modification are critical for the quality control of products. Improving reproducibility and scalability are also factors worth paying attention to when optimizing cell surface modification strategies.

## CONCLUSIONS AND PERSPECTIVES

Tiny living organisms including cells and microorganisms provide a paradigm shift for the development of therapeutic agents for cancer immunotherapy due to their versatile endogenous functions such as immunogenicity, targeting ability and long circulation. Despite the fact that numerous therapeutic systems based on eukaryotic cells, cell membrane vesicles, bacteria, OMVs, viruses and virus-like particles have been widely used to promote antitumor immune responses, they remain stalled in preclinical research or are aborted in clinical trials [136,137]. Besides the difficulty of preparation and quality control, the inconsistency between *in vivo* safety and efficacy is the biggest development bottleneck.

Advances in biotechnology have broken down the barriers of original natural materials, providing technical support for shaping bio-derived systems according to different therapeutic needs and disease characteristics. The means currently used to modify organisms include biological, chemical and physical dimensions as well as their integrations [25,106,119]. Functional proteins introduced by gene engineering can be stably and persistently expressed and mimic endogenous analogues. However, with regard to scaled-up manufacture, new problems different from those in laboratory research have to be solved. For instance, the preparation complexity makes quality control and impurity separation more difficult, and unremoved gene transfection vectors may cause safety issues associated with genetic recombination. Covalently bonded modification is robust and the amount of coupled molecules can be controlled by adjusting the feed, but linking functional groups may affect the basic behavior of biological systems. Physical binding is easy but has poor stability and low controllable binding efficiency.

Engineered organism-derived systems improve the specificity, efficacy, spatiotemporal controllability and safety of immunotherapy by retaining the original advantages through multiple strategies aimed at two steps of the delivery process: reach and action. Equipping the DDS with small molecules or proteins that can target tumor cells or immune cells promotes ‘reach step’ by increasing drug accumulation at the target site and drug internalization by specific cells. As for the ‘action step’, introducing environmentally responsive genetic elements, chemical bonds and physical interaction forces can establish links between the environment, drug release and action, reducing off-target effects of the drug and improving safety. For instance, the construction of CAR-T cells with the synNotch toolkit and logic gates to recognize specific antigens and activate CAR expression enables tumor-specific killing [53]; the introduction of tumor-specific promoters into the genetic circuit avoids therapeutic protein leakage; enzyme- or acid-sensitive cell-derived delivery systems specifically activate prodrugs at tumor sites [138,139]. Inserting genetic circuits that respond to exogenous signals, such as small molecules or physical stimuli, can control the timing, space and intensity of the treatment. For instance, bacteria with lactose operons, temperature-sensitive promoters, or SLCs can release immunomodulators in response to IPTG, temperature or population density to avoid over-activating the immune system and cytokine storm [72,75,77]. Modification with immune activators would help to break immune tolerance. For instance, immune checkpoint blockers expressed on cells or cell membrane vesicles reversed the immunosuppressive TME [36]; the construction of cell-derived vaccines co-expressing tumor antigens and co-stimulatory molecules activated antitumor immunity [26]; endowing cells, bacteria and viruses with cytokine-secreting ability promoted the recruitment and killing capacity of antitumor immune cells [5]; in addition, safety can be enhanced by deleting toxicity factors [30].

Artificial intelligence (AI) has the potential to process data at the genomic, protein and cellular levels, thereby contributing to the advancement of novel cell-engineered therapeutic approaches [140,141]. Depending on the target of action, AI can directly improve the development of cellular engineering by designing gene editing sequences for engineered cells and switches and logical gate structures for CARs, and screening cell surface functionalization motifs. In addition to assisting in modifying strategies, AI can also enhance the efficiency of cell engineering toolkits such as gene editing enzymes through directed evolution [142,143]. In the future, under powerful simulation and computational capabilities, researchers can hopefully achieve the construction of ‘top-down’ artificial cells. It is worth noting that, despite the potential of AI in bio-inspired therapeutic systems, the advancement of AI-assisted cell engineering is constrained by practical considerations, including the scarcity of accessible biological data and the heterogeneity of diverse samples.

New features present additional challenges. Despite promising results demonstrated in pre-clinical experiments and clinical trials, further research is needed to facilitate the bench-to-bedside translation of therapy technology based on engineered organisms [47]. First, culture, expansion and viability retention of living cell systems are critical issues during manufacture and storage. Clinical and regulatory work is underway to explore more standardized and automated manufacturing of cellular products to expedite the development of new cell-based therapies [144]. Therapeutic systems affect the life activities of treated bodies through various signaling pathways, while their vitality and persistence depend greatly on the *in vivo* environment. Therefore, the relationship between therapeutic systems and the body needs to be studied at the molecular, cellular and tissue levels with the assistance of multi-omics approaches. High-throughput screening and AI technologies can accelerate the development and optimization of DDSs. The integration of interdisciplinary approaches including immunology, oncology, synthetic biology and molecular biology will further boost the research and development of living-organism-derived systems for cancer immunotherapy.
